# Cross-border outbreak of listeriosis caused by cold-smoked salmon, revealed by integrated surveillance and whole genome sequencing (WGS), Denmark and France, 2015 to 2017

**DOI:** 10.2807/1560-7917.ES.2017.22.50.17-00762

**Published:** 2017-12-14

**Authors:** Susanne Schjørring, Sofie Gillesberg Lassen, Tenna Jensen, Alexandra Moura, Jette S Kjeldgaard, Luise Müller, Stine Thielke, Alexandre Leclercq, Mylene M Maury, Mathieu Tourdjman, Marie-Pierre Donguy, Marc Lecuit, Steen Ethelberg, Eva M Nielsen

**Affiliations:** 1 Department of Bacteria, Parasites and Fungi, Statens Serum Institut, Copenhagen, Denmark; 2 Department of Infectious Disease Epidemiology and Prevention, Statens Serum Institut, Copenhagen, Denmark; 3 The Danish Veterinary and Food Administration, Copenhagen, Denmark; 4 Institut Pasteur, National Reference Centre and WHO collaborating Center for Listeria, Biology of Infection Unit, Paris, France; 5 National Food Institute, Technical University of Denmark, Kgs. Lyngby, Denmark; 6 Santé Publique France, the French Public Health Agency, Saint-Maurice, France; 7 Ministry of Agriculture, and Forestry, Paris, France; 8 Paris Descartes University, Sorbonne Paris Cité, Division of Infectious Diseases, Necker-Enfants Malades University Hospital, Institut Imagine, F-75743 Paris, France

**Keywords:** listeriosis, surveillance, WGS, food-borne infections, Cross-border outbreak, cold-smoked salmon, whole genome sequencing

## Abstract

In August 2017, an outbreak of six listeriosis cases in Denmark was traced to cold-smoked salmon, using epidemiological investigations and whole-genome sequencing (WGS) analyses. Exchange of genome sequences allowed identification in France of a food isolate from a salmon-derived product and a human isolate from 2016 within the same cgMLST cluster as the Danish isolates (L2-SL8-ST8-CT771). The salmon product came from a third European Union country. WGS can rapidly link human cases and food isolates across Europe.

## Identification of the outbreak

In Denmark, on 23 August 2017, Statens Serum Institut (SSI) identified a genetic cluster of four human *Listeria monocytogenes* sequence type (ST) 8 isolates by core genome multilocus sequence typing (cgMLST) [1]. The allele calling was performed in BioNumerics (v7.6.2, Applied Maths, Belgium). We initiated an epidemiological investigation and notified the Danish Central Outbreak Management Group (collaboration between the Danish Veterinary and Food Administration (DVFA), the National Food Institute at the Technical University of Denmark (DTU) and SSI). On 25 August, two additional human isolates were found to belong to the same genetic cluster.

Raw sequence data of four outbreak isolates, SSI-AC382–5, are deposited at the European Nt Archive (ERS2039635-8).

### Case definition

A confirmed case was defined as a person clinically diagnosed with listeriosis after 1 January 2017 with laboratory-confirmed *L. monocytogenes* ST8 clustering using cgMLST (≤ 5 allelic distance, single linkage). Cases diagnosed before 1 January 2017 with an isolate belonging to this cluster were defined as probable cases. 

## Case description and food exposure of Danish patients

**Figure 1:**
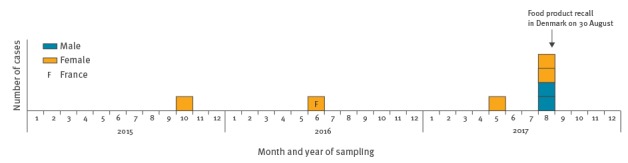
Number of probable and confirmed cases of listeriosis, by month and year of laboratory sample date, sex and country, Denmark and France, 2015–2017 (n = 7 cases)

As of 25 August 2017, the genetic cluster comprised six cases; five confirmed and one probable. Laboratory sample dates ranged from 25 October 2015 to 21 August 2017 (Figure 1). The age of the cases ranged from 59 to 96 years (median 80 years) and four were women. All patients had underlying illness and no travel history. One patient died within 30 days of diagnosis. Epidemiological investigations including a standard questionnaire on exposures showed that all five confirmed cases had consumed cold-smoked and/or cured salmon in the 30 days before disease onset. Four cases had bought the salmon in retail chain X. No other food-item was reported as consumed in high frequencies among cases. Epidemiological follow-up for the probable case did not include information on fish consumption.

Unless otherwise specified with F, cases are from Denmark.

## Food investigation and control measures

On 29 August 2017, a comparison between the human outbreak isolates and 16 *L. monocytogenes* ST8 food- and environmental isolates identified in Denmark from 2014 to August 2017 showed that the human isolates clustered with a food isolate from cold-smoked salmon, cut and packaged at company Y in Poland (zero to two allelic differences using cgMLST). *L. monocytogenes* had been detected on 31 July 2017 at levels of 110 CFU/g (threshold: 100 CFU/g) at the end of shelf life. The product was widely sold in Denmark and had been sampled by the DVFA in retail chain X, as part of a consumer exposure survey (i.e. analyses project on retail packages). Because the *L. monocytogenes* concentration had been just above the accepted limit and found at the end of the product shelf life a recall of this batch was not conducted. However, due to the positive finding, follow-up sampling had been performed on the 9 and 10 August 2017 from the central storage unit of retail chain X. *L. monocytogenes* had been isolated from two batches analysed before end of shelf life. In one sample from the same batches, which was also analysed at the end of the shelf life, on 28 August 2017 a *L. monocytogenes* level of 240 CFU/g was found. Isolates from the follow-up samples had zero to four allelic differences to the human outbreak isolates using cgMLST.

**Figure 2:**
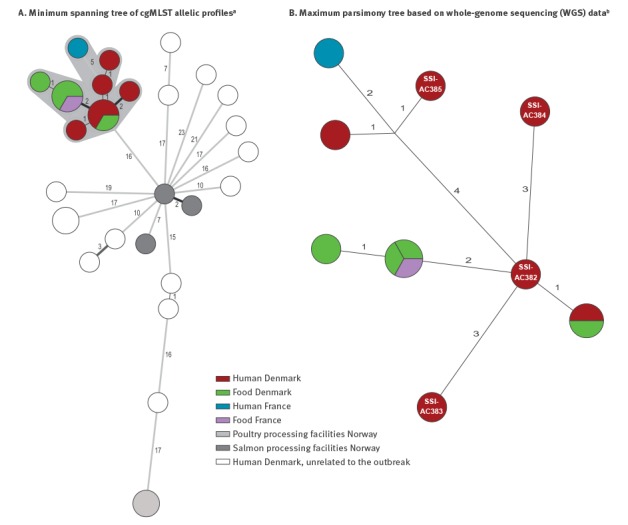
Whole genome sequencing based typing of *Listeria monocytogenes* ST8 isolates as part of of a cross-border listeriosis outbreak investigation, Denmark and France, 2015–2017

The human outbreak sequences were also compared to all *L. monocytogenes* ST8 genomes derived from clinical samples in Denmark from 2012 onwards. Although ST8 genomes from Danish patients in the period 2012–2017 showed high diversity, the outbreak isolates clearly formed a distinct cgMLST cluster with 16 allelic differences to the nearest isolates outside the genetic outbreak cluster and a maximum of nine allelic differences within the cluster (Figure 2a). We investigated the relatedness of outbreak isolates further by single-nucleotide polymorphisms (SNP) analysis performed by both SSI and DTU using two analysis pipelines: Northern Arizona SNP Pipeline (NASP) [2] and CSI Phylogeny version 1.4 from Center for Genomics Epidemiology (CGE), DTU [3] leading to the same conclusion. The SNP analysis showed a maximum of nine SNPs between any two isolates in the cluster (Figure 2b). The food isolate sampled in Denmark in July was identical by SNP analysis to one of the Danish patient outbreak isolates from August 2017.

cgMLST: core genome multilocus sequence typing; SSI: Statens Serum Institut.

The size of nodes corresponds to the number of isolates. Branch lengths represent the SNP difference.

^a^ Minimum spanning tree of cgMLST allelic profiles of 21 *L. monocytogenes* ST8 isolates from Danish patients in the period 2012–2017, 1 isolate from a probable French case 2016 and food isolates sampled in Denmark (4 isolates; July-August 2017) and France (1 isolate; September 2017) from products produced by company Y. The outbreak cluster is shaded in grey. Published sequences of ST8 isolates (2001–2013) from a study [17] on persistence in a poultry processing facility (SRR3099221, SRR3099222) and a salmon processing facility (SRR3099223, SRR3099224, SRR3099225) were included.

^b^ Maximum parsimony tree based on whole-genome sequencing (WGS) data showing single-nucleotide polymorphisms (SNPs) within the *L. monocytogenes* ST8 isolates from patients and food sources in the genetic outbreak cluster. One isolate (SSI -AC382, ERS2039635) from the cluster was used as the reference when using NASP.

On 30 August 2017, DVFA advised retail chain X to recall all cold-smoked salmon produced at company Y. This advice was based on the elevated number of *L. monocytogenes* (240 CFU/g) found in the product at the end of shelf-life and the link to the outbreak. Retail chain X voluntarily recalled both cold-smoked and cured salmon produced at company Y. As part of the recall procedure, retail chain X informed company Y on the situation. Information from company Y, provided by the Polish food authorities via the European Union Rapid Alert System for Food and Feed (RASFF), showed that the implicated batches were exclusively sold via retail chain X and only in Denmark.

## International enquiry and investigation in France

On 31 August 2017, the outbreak was notified internationally on different communication platforms: (i) the European Epidemic Intelligence Information System for food- and waterborne diseases (EPIS-FWD) reference UI-426, (ii) Early Warning and Response System (EWRS) notification reference EWRS20170831DK0001 and (iii) RASFF notification reference RASFF-2017.1319. The EPIS-FWD platform allowed sharing of files with assembled genomic sequence data. SSI distributed raw sequence data of four outbreak isolates (SSI-AC382-AC385) on a local ftp server at SSI.

The French National Reference Centre (NRC) for *Listeria* (Institut Pasteur, Paris), compared the sequences of the Danish human isolates against its database, using cgMLST as previously described [1,4]. A human isolate from a French resident belonged to the same cluster (L2-SL8-ST8-CT771) as the Danish isolates. This French probable case, a female patient in her mid-80s, was diagnosed in June 2016. Epidemiological investigations carried out by Santé Publique France were inconclusive, since food consumption history was not available at the time of diagnosis nor could information on travel to Denmark be retrieved, as the person had since died.

On 6 September 2017, an official control by the Ministry**of Economy was carried out at a French retailer where a kosher chilled cured salmon was sampled for analysis. The sample was contaminated with *L. monocytogenes* at the level of 460 CFU/g and the salmon producer was company Y. An isolate was sent to the French NRC for typing and showed to belong to the same cgMLST type as the Danish outbreak (Figure 2). Further investigations on the food product confirmed that it had not been further processed after production in Poland. The product was recalled and no human cases were linked to its consumption as of beginning of December 2017.

The other nine countries that replied to the EPIS-FWD UI-426 notification (Austria, Finland, Germany, Luxembourg, the Netherlands, Norway, Slovenia, Sweden, United Kingdom) did not report any human or food isolates linked to the Danish outbreak. However, after submission of this report, at the end of November, we were informed through EPIS about three genetically linked human isolates in Germany. 

## Discussion

Here we report on a listeriosis outbreak and highlight the value of rapidly comparing the genomes of human and food/environmental isolates at the national and international levels.

The fact that the contaminated salmon products identified in Denmark and France were from different batches suggests environmental contamination possibly at the production facility at company Y. It is too early to assess whether any measures taken at company Y have been effective in controlling the outbreak. However, experiences from previous investigations suggest that once *L. monocytogenes* is detected in one product, the whole production site should be subject to a thorough inspection, and sampling with special attention to all the possible contamination/cross contamination issues before implementing corrective measures [5,6]. Moreover, the risk for *L. monocytogenes* persistent strains in the production environment requires the close monitoring for several years to ensure the elimination of these [7,8].

Since WGS was introduced for routine surveillance in Denmark, a number of listeriosis outbreaks have been detected and solved, including outbreaks involving cold-smoked ready-to-eat sliced fish products [5]. The present investigation further reinforces the suspicion that ready-to-eat fish products are important sources of *L. monocytogenes* infections in Denmark, as well as in other countries.

Though only involving a low number of isolates, WGS *L. monocytogenes* surveillance and communication between countries allowed us to detect and rapidly solve this salmon-associated outbreak, leading to food product recall in two European countries. Compared with previous typing methods, WGS has a higher discriminatory power and the ability to determine genetic distance between isolates. The introduction of WGS for surveillance of food-borne infections has shown that it improves outbreak detection and facilitates outbreak investigations and likely helps reduce the number of infections [4,9-16]. The EPIS-FWD communication platforms allowed for the communication to link cases across borders. However, currently cross-border outbreaks are only detected when case numbers in at least one country exceed normal levels and are notified internationally. Therefore, a possible future system for easy exchange of and comparison of WGS data, e.g. by the use of an agreed cgMLST nomenclature, across borders will enable the identification of more dispersed outbreaks as well as cross-border links between food samples and human infections. This report highlights that by the application of cross-disciplinary and real-time cross-border comparison of WGS data, *L. monocytogenes* infections can be prevented and thereby providing safer food for at-risk groups such as the elderly, immunodeficient individuals and pregnant women.
